# 

**Published:** 1923-06

**Authors:** 


New Series. Vol. II. No. 21 June 1923 Price Sixpence

CONTENTS.

| | PAGE |
|---|---|
| Incipient Insanity | 221 |
| Smoke and Health | 222 |
| Notes and Comments | 223 |
| St. Bartholomew's Octocentenary | 227 |
| Panel Pay and Extended Services | 229 |
| Panel and Patient | 230 |
| A Medical Utopia | 230 |
| The Public Health: Interviews with Local Authorities.—Bradford | 231 |
| Hospital Progress and Finance | 233 |
| The Mental Treatment Bill: Interview with Sir F. Willis | 235 |
| Health Insurance Notes | 237 |
| British Hospital Association: The Sheffield Conference | 238 |
| Royal Society Congress | 241 |
| Scarborough Health Conference | 241 |
| Puerperal Insanity | 242 |
| Work of Medical Missions | 242 |
| Wasting Money on Neurotics | 243 |
| Correspondence | 244 |
| Reviews of Books | 245 |
| Echoes of the Month | 249 |
| Hospital and Health News | 250 |
| Recent Appointments | 252 |
| Public Health in Parliament | 252 |

